# Modulation of the Conformational Space of SARS‐CoV‐2 RNA Quadruplex RG‐1 by Cellular Components and the Amyloidogenic Peptides α‐Synuclein and hIAPP

**DOI:** 10.1002/chem.202104182

**Published:** 2022-01-05

**Authors:** Sanjib K. Mukherjee, Jim‐Marcel Knop, Roland Winter

**Affiliations:** ^1^ Physical Chemistry I - Biophysical Chemistry Department of Chemistry and Chemical Biology TU Dortmund University Otto-Hahn Street 4a 44227 Dortmund Germany

**Keywords:** α-synuclein, hIAPP, RNA G-quadruplex, SARS-CoV-2, single-molecule FRET

## Abstract

Given the emergence of the severe acute respiratory syndrome‐coronavirus‐2 (SARS‐CoV‐2), which particularly threatens older people with comorbidities such as diabetes mellitus and dementia, understanding the relationship between Covid‐19 and other diseases is an important factor for treatment. Possible targets for medical intervention include G‐quadruplexes (G4Qs) and their protein interaction partners. We investigated the stability and conformational space of the RG‐1 RNA‐G‐quadruplex of the SARS‐CoV‐2 N‐gene in the presence of salts, cosolutes, crowders and intrinsically disordered peptides, focusing on α‐Synuclein and the human islet amyloid polypeptide, which are involved in Parkinson's disease (PD) and type‐II diabetes mellitus (T2DM), respectively. We found that the conformational dynamics of the RG‐1 G4Q is strongly affected by the various solution conditions. Further, the amyloidogenic peptides were found to strongly modulate the conformational equilibrium of the RG‐1. Considerable changes are observed with respect to their interaction with human telomeric G4Qs, which adopt different topologies. These results may therefore shed more light on the relationship between PD as well as T2DM and the SARS‐CoV‐2 disease and their molecular underpinnings. Since dysregulation of G4Q formation by rationally designed targeting compounds affects the control of cellular processes, this study should contribute to the development of specific ligands for intervention.

## Graphical abstract

**Pre‐existing conditions** play a critical role in disease progression after infection with severe acute respiratory syndrome coronavirus 2. In this study, we sought to unravel the underlying mechanisms related to the interactions between peptides relevant to type II diabetes mellitus and Parkinson's disease and the G‐quadruplex‐forming sequence RG‐1 of the viral RNA.

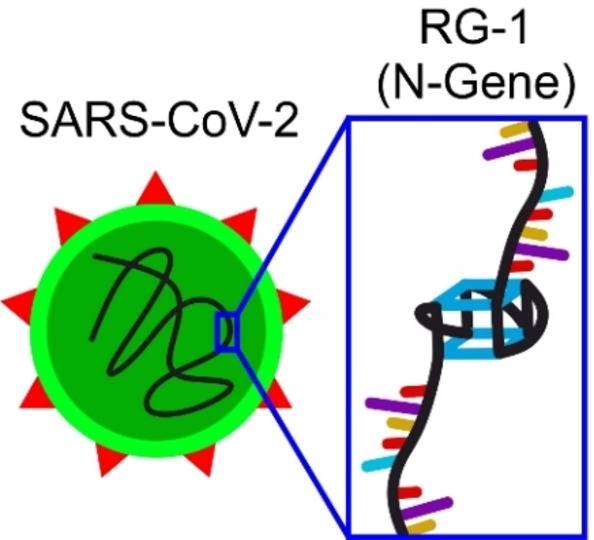

## Introduction

**Figure 1:**
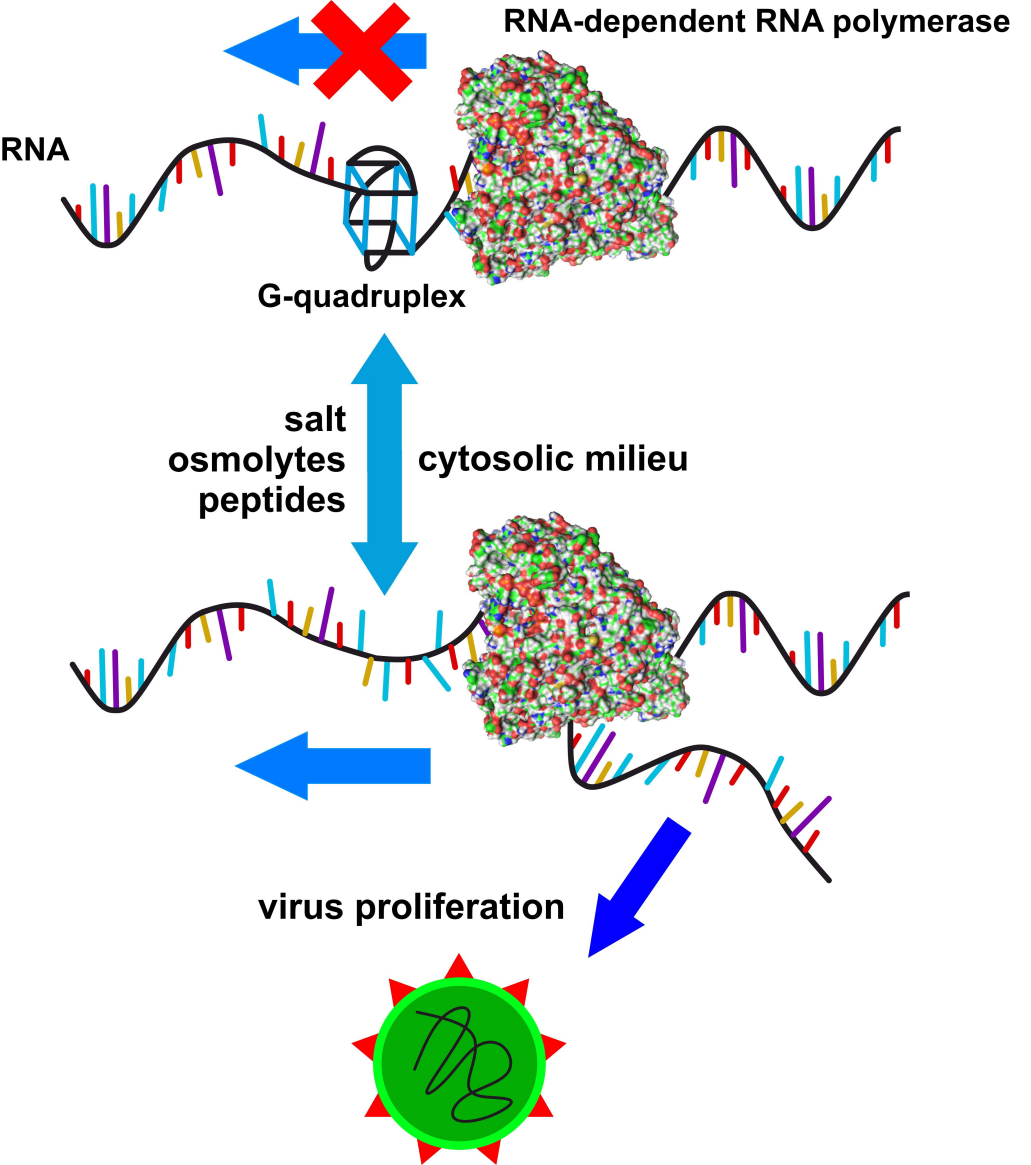
G‐quadruplex formation can decrease transcription and translation of viral RNA and therefore reduce viral proliferation. The formation and stability of the G‐quadruplex is highly dependent on its chemical environment, that is, the components of the cellular milieu, and on the presence of intrinsically disordered peptides.

The COVID‐19 pandemic is caused by an enveloped RNA coronavirus called severe acute respiratory syndrome‐coronavirus‐2 (SARS‐CoV‐2). Next to the respiratory tract, the COVID‐19 virus affects several other organs.[^1^, ^2^, ^3^] Currently, researchers are following three major approaches to develop therapeutics for fighting COVID‐19: vaccines, neutralizing antibodies, and antiviral drugs, the main focus being on viral proteases and their inhibitors.[^4^, ^5^, ^6^, ^7^, ^8^] Recently, non‐canonical nucleic acids structures, such as G‐quadruplexes (G4Qs), have also been recognized as promising therapeutic targets.[^7^, ^8^, ^9^, ^10^, ^11^] The structure of G4Qs is characterized by the stacking of two or more planar arranges of G‐tetrads which are stabilized by lateral Hoogsteen‐type hydrogen bonds and by the coordination of a monovalent cation, such as K^+^ (Figure 1).^[11]^ Depending on the orientation of the G‐tetrades, the structure of G4Qs can be parallel (with four G‐tetrades in the same orientation), antiparallel (with two G‐tetrades in opposite orientation with respect to the other two), or hybrid (with one G‐tetrade in opposite orientation with respect to the other three) and further distinguished by their loop position (for example, antiparallel basket or chair conformation as well as different types of hybrid structures).^[11]^ DNA/RNA polymerases coordinate their action with enzymes that unwind G4Qs, known as G4‐helicases. G4Qs have therefore received considerable attention over the last twenty years due to their involvement in the regulation of cellular processes including replication, transcription and translation.[^10^, ^11^, ^12^, ^13^, ^14^, ^15^] Dysregulation of G4Q formation and their binding proteins, which assist them in regulating the equilibrium between their structured and unstructured/unfolded forms, due to mutations or through the alteration of their stability by environmental factors (for example, by changes in intracellular solution conditions or by G4Q‐stabilization induced by a ligand), have been found to contribute to many human pathologies, including neurodegenerative diseases, cancer, and microbial infections.[^4^, ^5^, ^6^, ^7^]

In addition to eukaryotes and prokaryotes, G4Q motifs are also considered key elements in regulating the life cycle of viruses.[^4^, ^5^, ^6^, ^7^, ^8^, ^9^] Their G4Qs repress the expression of viral proteins, some of which play immunomodulatory roles by limiting antigen presentation to cytotoxic T cells, thereby allowing the virus to survive in infected cells without being recognized by the host immune system. Hence, G4Q‐specific compounds could be potential candidates for antiviral agents. Recently, it has been shown that G4Q‐forming sequences in SARS‐CoV‐2 can form G4Q structures in living cells.[^7^, ^9^] Hence, they would be novel targets for G4Q‐specific compounds that could exert antiviral activity. The G4Q‐forming sequence RG‐1 is located in the coding sequence region of the SARS‐CoV‐2 nucleocapsid phosphoprotein (N‐protein). In fact, the formation of RG‐1 G4Q has been shown to decrease the amount of N‐protein by inhibiting its translation. The RG‐1 quadruplex was found to consist of two G‐tetrad layers and to have a parallel G4Q structure, according to CD spectroscopy data.^[7]^ Given the central role of the N‐protein in controlling virus assembly and replication, RG‐1 G4 may be a promising therapeutic target for SARS‐CoV‐2.^[7]^ A prerequisite for the development of G4Q‐related antiviral drugs against COVID‐19 is a detailed knowledge of the structure and stability of the G4Q.

It is well known that the structure and thus the function of RNAs such as RNA‐G4Qs are very susceptible to changes in their environment, that is, they respond sensitively to the presence of salts, osmolytes, crowding agents, and to the binding of proteins, which are also abundant in the biological cell.[^16^, ^17^, ^18^, ^19^, ^20^, ^21^, ^22^, ^23^] In general, the stability of quadruplex structures is determined by H‐bonds between nucleobases, π‐stacking interactions between neighboring base pairs, counterion condensation at the phosphate backbone by cations, hydration changes, and by changes in conformational entropy.^[11]^ Recently, it has been observed that the stability of the G4Qs depends also on the number of G‐quartets. For example, the polyethylene glycol polymeric crowding agent PEG200 was found to stabilize RNA‐G4Qs with three or four G‐quartets but did not change the stability of G4Qs with two G‐quartets.^[11]^

In this study, we explored the effects of central environmental factors, including salts, osmolytes, crowding agents and intrinsically disordered peptides (IDPs), on the conformational landscape of the RG‐1 G4Q of SARS‐CoV‐2. As IDPs we selected α‐Synuclein (α‐Syn) and the human islet amyloid polypeptide (hIAPP). α‐Syn regulates neurotransmitter vesicle cycling, but is, under pathological conditions, closely associated with Parkinson's disease (PD). PD results from abnormal aggregation of α‐Syn, and these aggregates are found primarily in Lewy bodies, the hallmark of PD.[^24^, ^25^, ^26^] Interestingly, SARS‐CoV‐2 has also been found in neurons in different brain regions.[^26^, ^27^, ^28^, ^29^] Recent reports of cases of Parkinson's disease in relatively young patients after a SARS‐CoV‐2 infection suggest that there may be a link between SARS‐CoV2‐infections and the development of PD.[^29^, ^30^, ^31^] In fact, multiple indications of a relation between amyloidoses and viral infections have been reported in the past.^[31]^ The human α‐Syn protein has 140 amino acid residues and consists of three distinct regions, which include an amphipathic N‐terminal domain, a central hydrophobic region (the non‐Aβ component (NAC) region), and a highly negatively charged proline‐rich C‐terminal domain. The conformation of the polypeptide is strongly affected by cosolutes and lipid membranes, and α‐Syn has also a high propensity to interact with DNAs.[^32^, ^33^, ^34^, ^35^, ^36^, ^37^, ^38^, ^39^, ^40^, ^41^] hIAPP (amylin) is a 37 amino acid hormone that is associated with the progression of type II diabetes mellitus (T2DM).[^42^, ^43^, ^44^] The peptide hormone misfolds to form amyloid deposits in and around the pancreatic islet β‐cells that synthesize both insulin and hIAPP, leading to a decrease in β‐cell mass in patients with the disease. Recent findings suggest that there is also a strong correlation between T2DM and SARS‐CoV‐2 diseases.[^45^, ^46^, ^47^] Therefore, studying the conformation of the SARS‐CoV‐2 RNA‐G4Qs in the presence of α‐Syn and hIAPP is of immense importance, as interaction with these disordered peptides can significantly affect replication and transcription along with causing genetic instability in host cells.

## Results and Discussion

### Effect of salts, crowding and cosolvents on the conformation of the SARS‐CoV‐2 RG1‐RNA quadruplex

**Figure 2:**
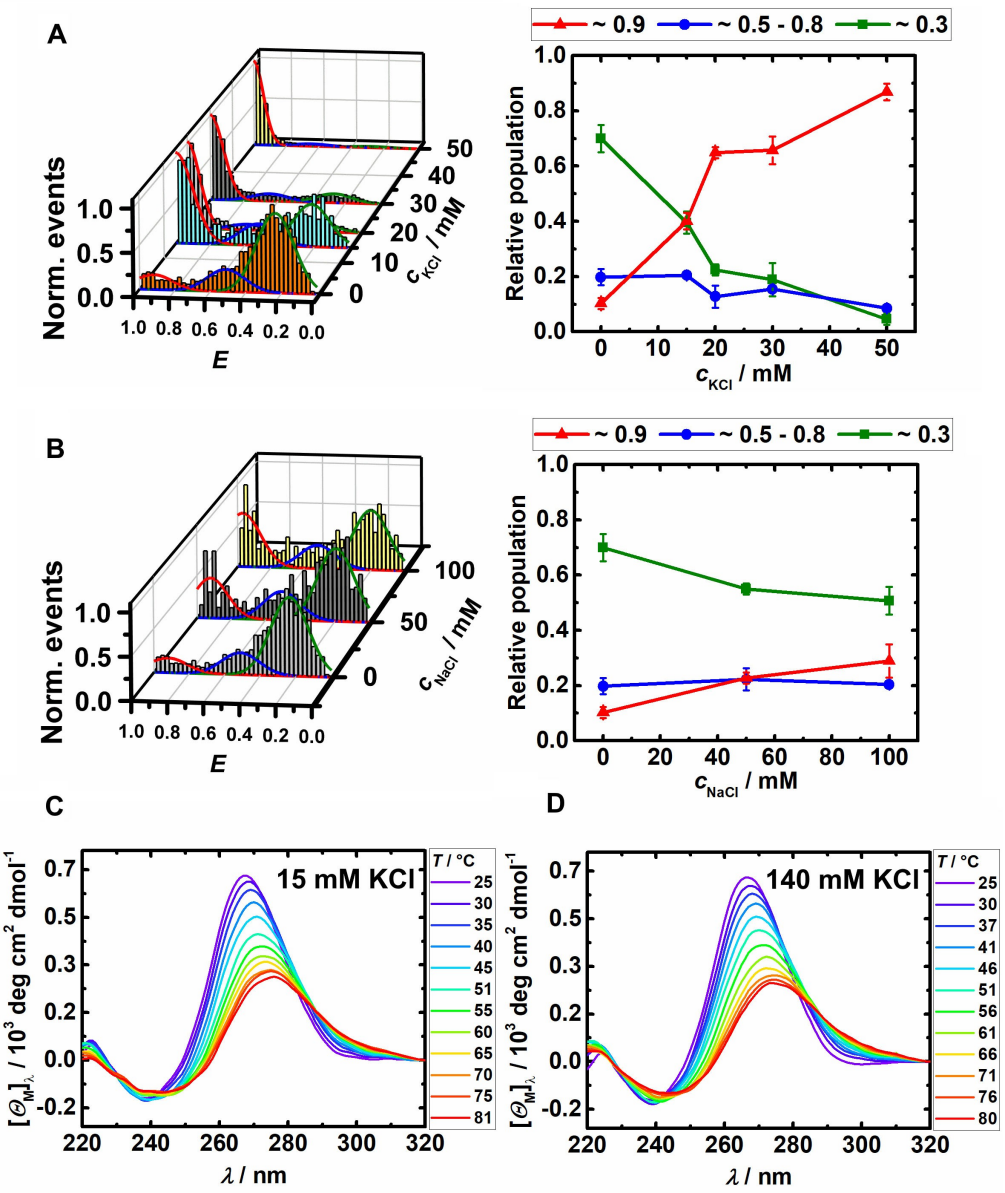
FRET efficiency and relative population of conformational states of the elongated and labeled RG‐1 (∼100 pM) RNA in 20 mM TrisHCl‐buffer at pH 7.5 and 25 °C at different A) KCl and B) NaCl concentrations. Temperature dependent CD‐spectra of the unlabeled sequence (20 μM) in 20 mM Na_x_H_x_PO_4_‐buffer, pH 7.5, with C) 15 mM and D) 140 mM KCl.

Since the main objective of this study was to investigate the effects of various environmental factors on the conformational landscape of the RG‐1 RNA quadruplex, we performed conformation‐sensitive single‐molecule Förster resonance energy transfer (smFRET) experiments. This method avoids ensemble averaging and enabled us to elucidate the structure and conformational dynamics of the quadruplex and how the equilibrium between conformational substates is affected by the different solution conditions.[^48^, ^49^] The peaks in the FRET efficiency histograms refer to conformations with different spatial separations, *R*, of the two attached dyes and thus different FRET efficiencies, *E*, because *E*=*R*_0_^6^ ⋅ (*R*_0_^6^+*R* ^6^)^−1^.[^21^, ^48^, ^49^] The Förster radius, *R*_0_, is the distance at which 50 % of the excited donor molecules will be deactivated (*R*_0_=6.5 nm for the fluorophores used, Atto 550 and Atto 647N). As it is well known that monovalent cations play an important role in the stability of the G4Qs, smFRET measurements were carried out in the presence of monovalent (K^+^, Na^+^) salts to quantify their effect on the conformational transitions of the RG‐1 SARS‐CoV‐2 RNA. Of note, in diseased cells such as those of cancer and neurodegenerative diseases, the concentration of intracellular ions is typically changed by overexpression (or inactivation) of disease specific ion channel proteins. We observed that the stability of the RG‐1 SARS‐CoV‐2 RNA‐G4Q is highly susceptible to the concentration of monovalent K^+^. In the absence of K^+^ cations (pure buffer solution), two peaks were observed in the FRET histograms, located at *E*≈0.3, representing the unfolded conformation) and at *E*≈0.5–0.8, representing a metastable or partially folded conformation, respectively (Figure 2A). At and beyond 15 mM KCl, a further conformation appeared, located at *E*≈0.9 in the histogram, which represents the folded conformation of the RNA G4Q. Conformational switching from open to closed states was observed between 0 and 30 mM KCl. At high K^+^ concentrations (140 mM KCl), only the fully folded conformation is retained (Figure 2A).

Complementary CD measurements at 15 mM KCl showed a positive band around 267 nm and a negative peak close to 240 nm, with no significant changes at higher salt concentrations (140 mM KCl), which can be attributed to a parallel topology of the G4Q (Figure 2C and D) and is similar to the CD spectrum of the core structure of the G4Q.^[7]^ Unfolding of the RG‐1 RNA‐G4Q at high temperature as observed in the CD measurements was also observed by corresponding smFRET measurements of the elongated RG‐1 G4Q (Figure S2). Interestingly, the Na^+^ cation was found to be unable to stabilize the folded conformation of the RG‐1 RNA‐G4Q (Figure 2B), even at 100 mM concentration. Conversely, the human telomeric (htel) DNA G‐quadruplex adopts a folded antiparallel topology in Na^+^ salt solution.^[49]^

Various factors contribute to the stability of G‐quadruplexes and determine their folding topology, including π‐stacking interactions, H‐bonding, cation binding, and hydration changes.[^11^, ^50^, ^51^] Since the stacking interactions partly account for the net energetic gain, increasing the number of quartets is energetically favorable and provides more stability.^[51]^ Along with that, stacking of other non‐tetrad bases can also contribute to the overall stabilization of the G4Q structure.^[51]^ Recent studies revealed that RG‐1 G4Q exhibits a parallel quadruplex topology which is composed of two superimposed tetrads and a flexible peripheral loop composed of adenines and cytosines, serving as a linker to the guanines involved in tetrad formation.[^7^, ^52^] Cation coordination is another essential factor in the stabilization of G4Qs.^[53]^ Cations bind to the negatively charged phosphates groups of the G4Qs both through nonspecific interactions and through specific (site‐bound) coordination to the O‐6 lone‐pair electrons of the guanines of the tetrades after loss of their hydration sphere. Binding of the cations to the negatively charged phosphate backbone reduces the electrostatic repulsions and thus provides stability to the folded conformation.[^54^, ^55^] As the ionic radius of K^+^ is larger (1.33 Å) compared to Na^+^ (0.95 Å), we may assume that Na^+^ should be small enough to be coordinated within the plane of a G‐quartet. Nevertheless, the free energy of binding to the RG‐1 RNA quadruplex seems to be much lower for K^+^, promoting the formation of a stable parallel‐folded G‐quadruplex beyond 15 mM KCl. For comparison, the htel telomeric DNA quadruplex adopts a hybrid‐stranded conformation in the presence of K^+^ and an antiparallel basket structure in the presence of Na^+^.[^47^, ^55^] The htel DNA G4Q consists of three superimposed tetrads. Therefore, we can assume that the binding of Na^+^ by an additional stacking interaction across the third tetrade provides sufficient stability, which is not possible in the case of the RG‐1 G4Q.

The intracellular environment is crowded with biomolecules that occupy a significant portion (up to 30 %–40 %) of the cellular volume, which is expected to affect the free energy and conformational landscape of biomacromolecules.[^21^, ^22^, ^23^, ^56^, ^57^, ^58^] We used 30 wt % Ficoll, a polysaccharide of about 5 nm size, to mimic cellular macromolecular crowding conditions. Figure S3 shows that crowding stabilizes the folded conformation of the RG‐1 G4Q. This effect can be understood invoking an entropy‐driven excluded volume effect. Due to the reduction of the solvent accessible surface area in the folded state, the equilibrium shifts toward the folded quadruplex structure upon addition of the crowding agent.

**Figure 3:**
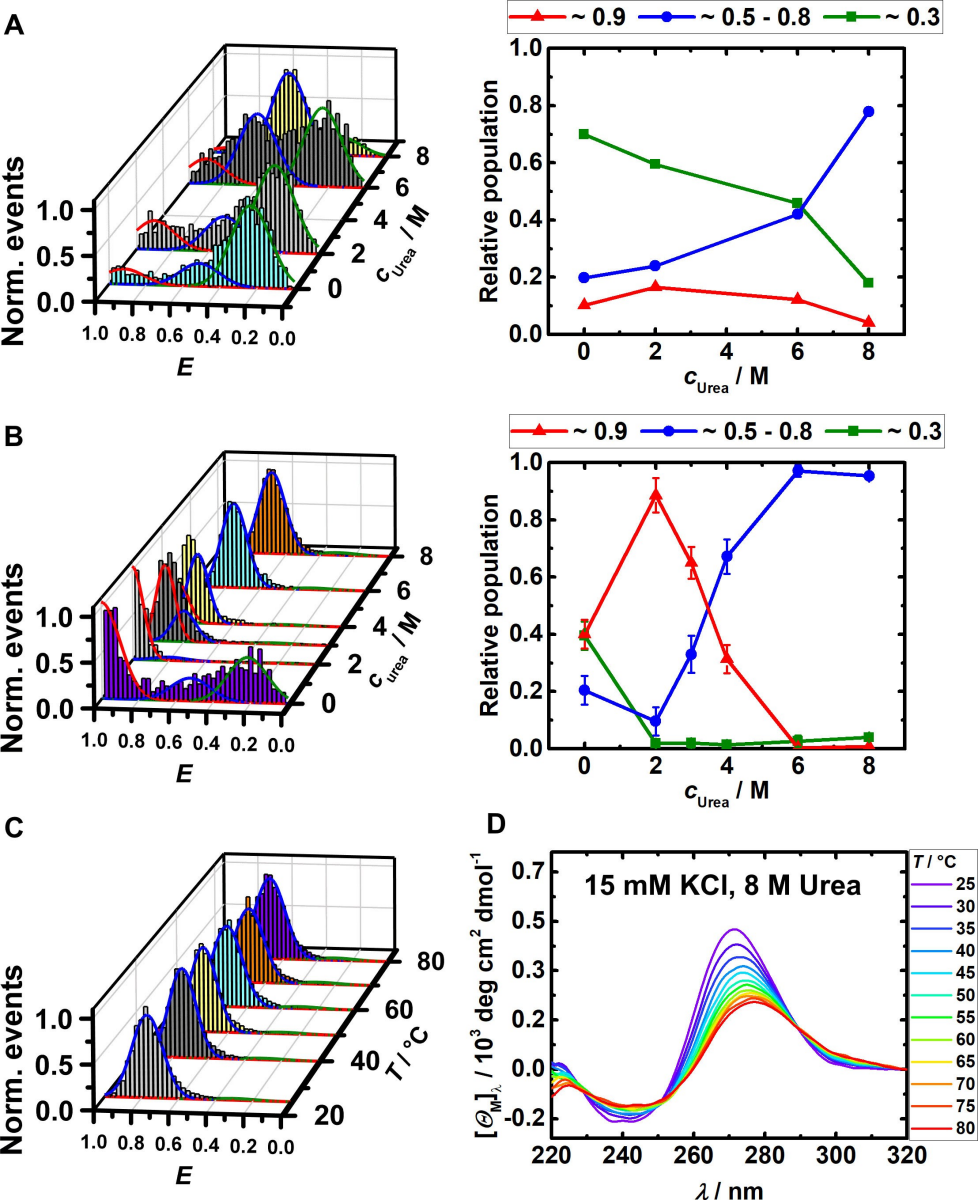
A and B) FRET efficiency and relative conformational population of the elongated and labeled RG‐1 RNA (∼100 pM) in 20 mM TrisHCl buffer at pH 7.5 and 25 °C with increasing urea concentration. Samples A) contained no salt, samples B) contained 15 mM KCl. C) shows the temperature dependent FRET efficiency histograms with 15 mM KCl and 8 M urea. D) depicts the corresponding temperature dependent CD spectra (20 μM RNA).

As a representative of a chaotropic cellular cosolute that generally interacts nonspecifically and unfavorably with proteins and nucleic acids (NAs), urea is a good choice. Urea is a natural organic cosolute with implications in maintaining osmotic pressure in cells and is present in rather large concentrations in various living organisms.^[59]^ We observed that urea strongly modulates the equilibrium between different RG‐1 G4Q conformations. Unexpectedly, in buffer with 15 mM KCl, urea concentrations beyond 2 M shifts the equilibrium towards the folded state (Figure 3B). At concentrations of 3–4 M urea, co‐existence of various conformations was observed in the smFRET histograms. We can tentatively assign the peak at *E*≈0.9 in the *E*‐histograms to a parallel folded conformation, the peak at *E*≈0.5–0.8 to a partially folded conformation, and that at *E*≈0.3 to the unfolded state structure. At high urea concentrations (6–8 M), a single conformation is mainly seen in the FRET histogram only, which can be attributed to a partially folded state. The partially folded conformer is steadily stabilized upon increasing the urea concentrations, reaching population values around 80 %. No drastic changes are seen in the CD spectrum of the partially folded state compared to the folded structure. For example, the CD spectrum of RG‐1 G4Q in 15 mM K^+^ and 8 M urea at room temperature showed a positive CD band around 270 nm, which is little red‐shifted compared to the pure buffer solution (15 mM K^+^, Figure 2C), along with a reduced amplitude and a negative peak close to 240 nm, implying that RG‐1 adopts a partially unfolded topology in accordance with the smFRET measurements that showed a single *E*‐distribution peak located at *E*≈0.8 (Figure 3C).

An increase in temperature lead to a further conformational change of RG 1 G4Q as manifested in a redshift and decrease in the amplitude of the positive CD peak, indicating unfolding. The combined CD and smFRET data at ambient temperature suggest that high urea concentrations cause the RG‐1 quadruplex topology to transform into a structure in which only the overhang conformation is altered, while the core of the RG‐1 G4Q structure remains essentially unchanged. Heat‐induced denaturation, as visible in the CD‐spectra at high urea concentration (Figure 3D), results in a small shift of the FRET efficiency only, from *E*≈0.8 to *E*≈0.7 (Figure 3C), indicating that unfolding of the core structure at temperatures above ∼50 °C keeps the dyes still in close proximity.

It is generally expected that urea leads to favorable interactions with the rings and functional groups of nucleic acid bases when becoming exposed to the solvent in the unfolded state.[^59^, ^60^] However, the mechanism that urea exerts on the stability of G4Qs is still not well understood. Moore and coworkers showed that the structure of a four‐stranded RNA‐quadruplex remains stable even at 8 M urea concentration.^[61]^ A pronounced accumulation of urea molecules at short distances around a DNA G‐quadruplex was seen in a study of Smiatek et al.^[62]^ Such accumulation of urea around the G4Q surface implies that water molecules are mainly replaced by urea. We can therefore assume that in the RG‐1 G4Q the number of hydrogen bonds between the G4Q and urea predominates over RNA‐water interactions at high concentrations of urea, but this does not lead to a significant destabilization of the G4Q structure, but only stabilizes a partially folded state, most likely through interactions with the loop structure. The unfolding of the core structure of the G4Q is observed at high temperatures. However, the unfolded state is more structured in the presence of urea than the heat‐induced state in pure buffer.

### Effect of disordered amyloidogenic peptides on the conformation of RG‐1 RNA G4Q

**Figure 4:**
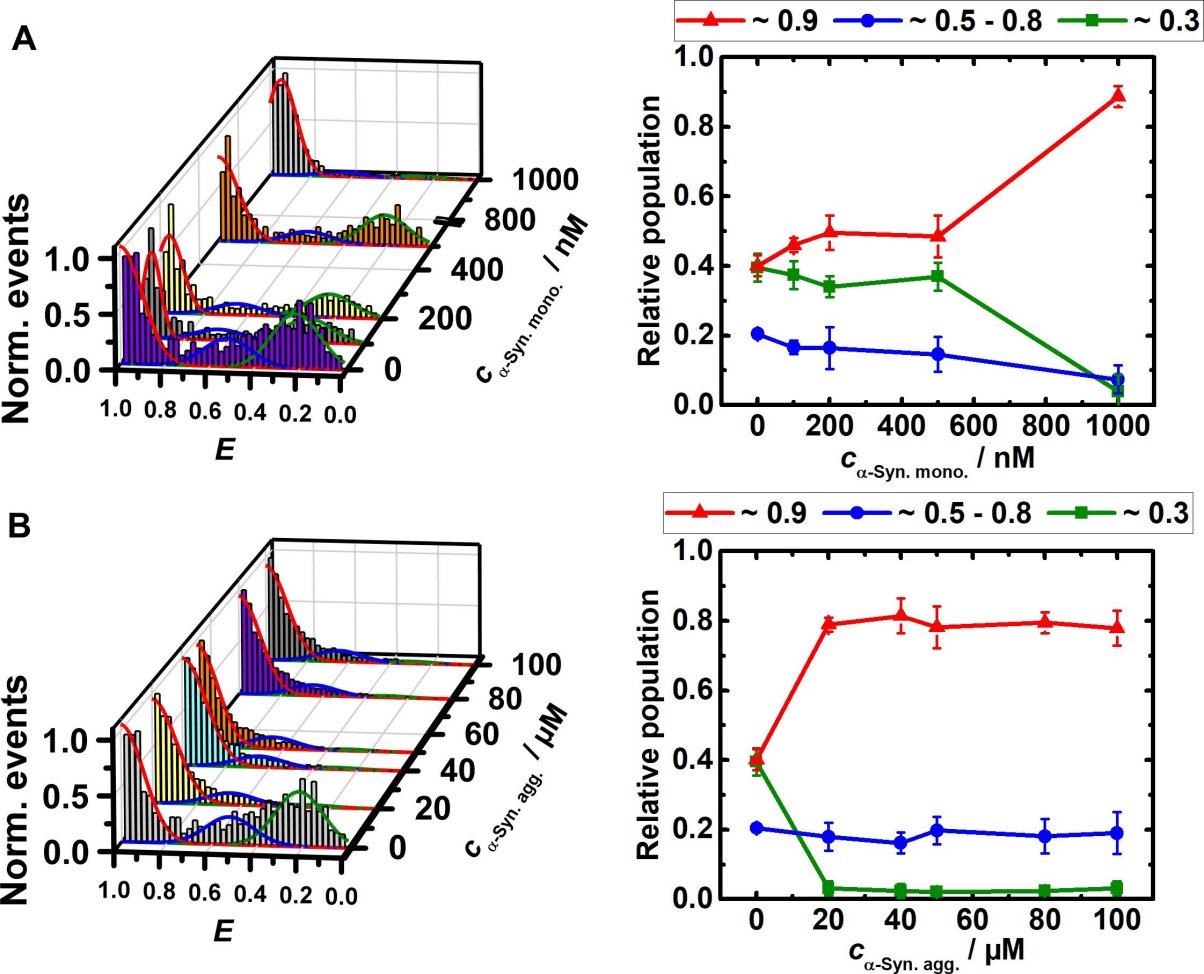
FRET efficiency and relative conformational population of the elongated and labeled RG‐1 RNA (∼100 pM) in 15 mM KCl, 20 mM TrisHCl buffer at pH 7.5 and 25 °C with increasing concentration of A) α‐Syn monomers and B) α‐Syn aggregates.

Intrinsically disordered proteins that bind to nucleic acids with high affinity are able to modulate the conformational dynamics of nucleic acids in a cellular environment and may hence have significant biological effects on gene expression and regulation patterns. We observed that the conformation of the SARS‐CoV 2 RG‐1 RNA G4Q is very susceptible to the presence of the amyloidogenic disordered peptides α‐Syn and hIAPP. As clearly seen in Figure 4, monomeric α‐Syn facilitates folding of the RG‐1 G4Q in the presence of 15 mM KCl already at sub‐μM concentrations, probably by acting as a macromolecular counterion that effectively reduces charge repulsion of the phosphate backbone of the nucleic acid. In the absence of α‐Syn, the RG‐1 G4Q adopts folded and unfolded conformations to a similar extent (∼40 %), along with smaller amounts of partially unfolded conformations (∼20 %, Figure 4A). With increasing concentration of monomeric α‐Syn (100 nM to 1 μM), the amount of folded RG‐1 G4Q conformers increases to ∼90 %. Remarkably, the conformational switching occurs already in the nanomolar concentration range. Increasing the α‐Syn concentration from 1 μM up to 100 μM has no further effect (Figure S4). In the presence of aggregated α‐Syn, a similar stabilization of the folded RG‐1 G4Q structure was observed. A completely different scenario was found in the case of human telomeric DNA G4Q.^[49]^ The conformation of the htel G4Q was largely unaffected by monomeric α‐Syn even at very high concentrations (200 μM). The antiparallel conformation of the htel G4Q gradually shifted towards a more unfolded conformation in the presence of oligomeric species of α‐Syn, but not in the presence of monomeric α‐Syn.

**Figure 5:**
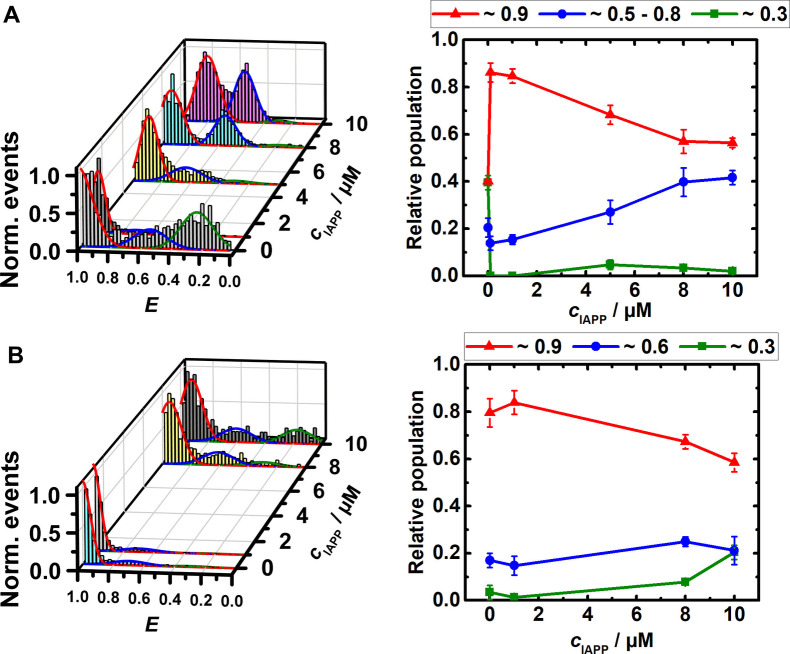
FRET efficiency and relative conformational population of the elongated and labeled RG‐1 RNA (∼100 pM) in 15 mM KCl, 20 mM TrisHCl buffer at pH 7.5 and 25 °C with increasing concentration of A) hIAPP and, for comparison, of the htel G4Q used in a previous study^[49]^ at the same solution conditions with increasing concentration of B) hIAPP.

Owing to the correlation found between type II diabetes and the SARS‐CoV‐2 disease, we may expect an effect of hIAPP on the replication and translation of the virus RNA.[^45^, ^46^, ^47^] Hence we studied also the interaction between hIAPP and the RG‐1 G4Q from the SARS‐CoV‐2 N‐gene. The smFRET data demonstrate that the conformation of RG‐1 G4Q is also strongly affected by hIAPP. Figure 5(A) shows that the equilibrium between unfolded and folded conformers of RG‐1 G4Q is strongly shifted to the folded state already at low hIAPP concentrations (0.1–1 μM). Moreover, a partially folded conformation (located at *E*≈0.5–0.6) is populated at higher hIAPP concentrations (5–10 μM). For comparison, the conformation of htel DNA‐G4Q was found to be less affected by the hIAPP (Figure 5B). Upon addition of 8 μM hIAPP, a small change from the antiparallel conformation (*E*≈0.9) to the hybrid topology (*E*≈0.6) was observed. In the presence of 10 μM IAPP, also a small amount of unfolded DNA (*E*≈0.3) was detected.

Protein‐RNA interactions are a vital mechanism inside the cell to perform RNA synthesis, translation and degradation.^[63]^ In general, protein interactions with nucleic acids are based on a series of molecular contacts, including hydrogen bonding (direct of mediated by water molecules), ionic/dipolar interactions, π‐interactions, van der Waals and hydrophobic interactions. Although some amino acid residues interact specifically with NAs,^[64]^ the diversity of structural motifs in proteins and NAs makes it difficult to comprehend the precise mechanism of protein‐NA interactions at the molecular level.[^65^, ^66^, ^67^, ^68^, ^69^] Reynaldo and coworkers showed that RNA binding occurs either through an α‐helix or loop of the protein or some amino acids involved in a β‐sheet structure which potentially interact with unpaired RNA bases.^[67]^ NMR measurements showed that a 12‐mer RNA aptamer forms a quadruplex structure capable of binding prion protein (PrP) peptides from the PrP′s N‐terminal region and blocks the structural conversion of PrP through both specific and nonspecific interactions.^[67]^

α‐Syn is a natively disordered protein that is positively charged (+4) at the N‐terminus and contains mainly lysine residues that can interact with the negatively charged phosphate groups of the nucleic acid, along with other amino acid residues that nonspecifically interact with the nucleobases of the RNA‐G4Q. α‐Syn has 17 glutamate residues along with 3 asparagines and 6 glutamines. Earlier reports suggested that lysine and arginine strongly favor interaction with guanine bases and largely account for the abundance of hydrogen bonding interactions with the bases.^[65]^ On the contrary, glutamine and asparagines are able to form a large number of hydrogen bonds with adenine bases, and glutamate is responsible for a maximum number of hydrogen bonds with cytosine. In addition, glutamate strongly interacts with cytosine through van der Waals interactions. Since RG‐1 G4Q has a flexible peripheral loop composed of adenines and cytosines, such interactions would help stabilize the folded state of the RG‐1 G4Q in the presence of α‐Syn. In the case of hIAPP, only 6 asparagines and 1 glutamine residue are present and able to form hydrogen bonds with adenine, and no additional stability is gained by other interactions. This might explain stabilization of a partially folded conformation at high concentrations of hIAPP, only.

## Conclusion

In recent years, G‐quadruplexes have received considerable attention as therapeutic targets for chemical intervention in biological functions. Since modulation of the stability and conformation of G4Qs has been found to play a significant role in many human pathological and neurodegenerative diseases, the underlying mechanisms need to be explored to be able to target G4Qs. We have shown that the stability and conformational landscape of the RNA G4Q‐forming sequence RG‐1, which is located in the coding sequence region of the SARS‐CoV‐2 nucleocapsid phosphoprotein (N), is broadly affected by salt, osmolyte, crowding and intrinsically disordered peptides.

**Figure 6:**
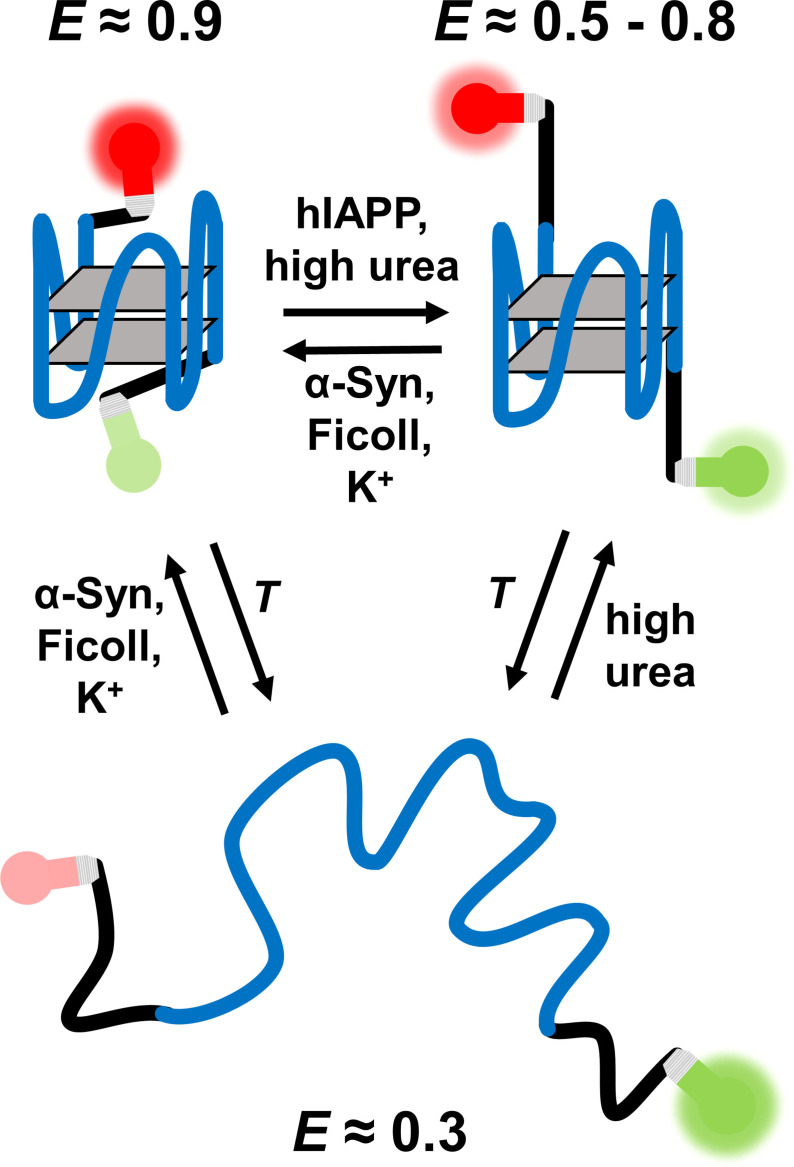
Schematic representation of the different effects salt cations, urea, crowder and the IDPs α‐Syn and hIAPP impose on the structure of the RG‐1 RNA G4Q. The blue line represents the G4Q forming NA, while the black lines represent the additional nucleotides. The gray planes are mimicking the G‐tetrads and the bulbs represent the fluorophores Atto 550 (green) and Atto 647N (red) used in the smFRET study.

Figure 6 gives an overview of the results obtained. Further, we have seen that significant differences exist between the stability and topology of the RG‐1 RNA‐G4Q and the human telomeric G4Q, providing novel insights into the mechanisms dictating the conformational preference of the different quadruplex structures. α‐Synuclein, the crowder Ficoll and K^+^ cations stimulate formation of the fully folded structure, while hIAPP as well as high concentrations of urea induce a partially folded conformation of the RG‐1 RNA‐G4Q. Only high temperature leads to full unfolding of RG‐1. The interaction with salts, cosolutes, crowders and peptides are factors that affect the conformational dynamics and stability of the G4Qs and helped us explore the overall conformational landscape of the different quadruplexes accessible. Since dysregulation of G4Q formation by rationally designed targeting compounds is detrimental in regulating cellular processes, this knowledge should be helpful in designing specific ligands for intervention. Next to the number of tetrades that determines the stability of the G4Qs, the loop sequences are unique and not only provide a structural distinction responsible for the diverse topologies observed, but may also have the ability to specifically interact with designer ligands.

To date, the link between SARS‐CoV‐2 and the metabolic and pathological diseases observed is not well understood. The formation of RG‐1 G4Q has been shown to reduce levels of N‐protein by inhibiting its translation, hence might be a promising therapeutic target to fight SARS‐CoV‐2. We have seen that both amyloidogenic peptides, α‐Syn and hIAPP, affect the conformational equilibrium between folded and unfolded states, to a different extent, however, in a sequence‐specific way. Considerable changes are observed with respect to their interaction with htel G4Qs. hIAPP stabilized a partially folded state of the RG‐1 RNA‐G4Q, whereas α‐Syn strongly stabilized the folded state of the quadruplex. Since G‐quadruplexes and their interaction with regulatory proteins play an important role as on/off‐switches that modulate polymerase activity, the interaction with these amyloidogenic peptides may alter expression profiles of disease‐modifying genes. Therefore, these results may shed more light on the relationship between PD and T2DM and the SARS‐CoV‐2 disease and its molecular basis.

## Experimental Section

**Materials**: Ficoll 70 was purchased from Carl Roth GmbH+Co. KG (Karlsruhe, Germany) and was used as received without further purification. Human IAPP (islet amyloid polypeptide or amylin) was purchased from GenScript (Leiden, Netherland). Before use, the IAPP peptide was dissolved in 1,1,1,3,3,3‐hexafluoro‐2‐propanol (HFIP) and incubated for at least 30 min to ensure an aggregate‐ and seed‐free sample. The buffer solution used in the measurements contains 20 mM Tris‐HCl (pH 7.4) and was filtered by a 0.45 μm sterile Whatman Puradisc 30 syringe filter. The RNA and DNA sequences with and without fluorophore labels were purchased from biomers (Ulm, Germany). For the exact sequences, please refer to the Supporting Information. The expression and purification of α‐synuclein was carried out as described before[^70^, ^71^] and is also described in the Supporting Information.

**Single‐molecule FRET (smFRET) measurements and data anaylsis**: SmFRET measurements were carried out using a confocal fluorescence microscope (MicroTime 200, PicoQuant) under freely diffusing conditions. The pulsed interleaved excitation (PIE) FRET technique was used to separate dually labeled from singly labeled species.^[72]^ Briefly, in the PIE FRET technique, both the donor and acceptor are alternatively excited by the laser pulse. First, a laser pulse of suitable wavelength excites the donor and then another laser pulse excites the acceptor independently from FRET after a certain time delay (50 ns, 20 MHz repetition rate), allowing us to calculate the photon stoichiometry, *S*, which is the ratio of photons emitted after donor excitation and the sum of total photons emitted after donor and direct acceptor excitation. For a donor‐only species, *S*=1, and for an acceptor‐species only, *S*=0. A green laser pulse at 560 nm (LDH series, PicoQuant) and a red laser pulse at 635 nm (LDH series, PicoQuant) were used to excite the donor Atto 550 and acceptor Atto 647N, respectively. A quad band dichroic mirror (ZT 405/488/561/640, Chroma) was used to reflect both the green and red laser light to the entrance port of the fluorescence microscope. Donor and acceptor fluorescence signals were separated to two different detection channels, first by using a dichroic mirror (FF 650 Di01, Semrock), followed by band pass filters FF 01‐593/40 (Semrock) and FF 01‐676/29 (Semrock). Two SPCM‐AQR series single photon avalanche diodes (SPAD) were used as detection channels for the donor and acceptor fluorescence. Time‐correlated single photon counting (TCSPC) data were first analyzed with Multiparameter Fluorescence Detection (MFD)^[73]^ script from Symphotime 5.3.2.2 software. The resulting histograms were normalized for the highest number of events and fitted with a three Gaussian function using Matlab R2016a with a least squares fitting method. For determining the relative populations of conformers, the percentage of the integrals of the single Gaussian functions were calculated and plotted.

**CD measurements**: The topology of G‐quadruplexes can be determined by monitoring the positive and/or negative circular dichroism (CD) signals at specific wavelengths. The CD measurements were performed using a JASCO J‐715 spectropolarimeter in 20 mM Na_x_H_x_PO_4_ buffer at pH 7.5 in the presence of 15 mM or 140 mM KCl solution. The concentration of the RNA construct was 20 μM. The molar ellipticity was calculated using the strand concentration. The pathlength of the quartz‐glass cell was 1 mm.

## Conflict of interest

The authors declare no conflict of interest.

1

## Supporting information

As a service to our authors and readers, this journal provides supporting information supplied by the authors. Such materials are peer reviewed and may be re‐organized for online delivery, but are not copy‐edited or typeset. Technical support issues arising from supporting information (other than missing files) should be addressed to the authors.

Supporting Information

## Data Availability

The data that support the findings of this study are available from the corresponding author upon reasonable request.
