# Supplementary material for: Modulation of the Conformational Space of SARS‐CoV‐2 RNA Quadruplex RG‐1 by Cellular Components and the Amyloidogenic Peptides α‐Synuclein and hIAPP
Source: Chemistry. 2022 Jan 5;28(9):e202104182. doi: 10.1002/chem.202104182 (PMC9015630; doi:10.1002/chem.202104182)
Supplement: Supplementary file 1 — Supporting Information [file CHEM-28-0-s001.pdf]

Chemistry–A European Journal

Supporting Information

Modulation of the Conformational Space of SARS-CoV-2 RNA Quadruplex RG-1 by Cellular Components and the Amyloidogenic Peptides α-Synuclein and hIAPP

Sanjib K. Mukherjee, Jim-Marcel Knop, and Roland Winter*

Materials and sample preparation

Ficoll 70 was purchased from Carl Roth GmbH + Co. KG (Karlsruhe, Germany) and was used as received without further purification. Human IAPP (islet amyloid polypeptide or amylin) was purchased from GenScript (Leiden, Netherland). Before use, the IAPP peptide was dissolved in 1,1,1,3,3,3-hexafluoro-2-propanol (HFIP) and incubated for at least 30 min to ensure an aggregate- and seed-free sample. The buffer solution used in the measurements contains 20 mM Tris-HCl (pH 7.4) and was filtered by a 0.45 μm sterile Whatman Puradisc 30 syringe filter. The RNA and DNA sequences with and without fluorophore labels were purchased from biomers (Ulm, Germany).

Nucleic Acid Sequences

RNA sequence for the single-molecule FRET microscopy measurements:

Atto 550 -5´-CUA GAA UGG CUG GCA AUG GCG GUG AUG CU-3´-Atto 647N

RNA sequences for CD-spectroscopy:

5´-CUA GAA UGG CUG GCA AUG GCG GUG AUG CU-3´

5´-GGC UGG CAA UGG CGG-3´

DNA sequences for single-molecule FRET microscopy:

5´-TGG GGA CGG CGA CGG A GGG TTA GGG TTA GGG TTA GGG X-3´
with X = T-Atto 550

5´-CCG YCG CCG TCC CCA-3´
with Y = Atto 647N

The annealing of the two DNA strands for the single-molecule FRET microscopy was carried out as described earlier in Knop, J.-M.; Patra, S.; Harish, B.; Royer, C. A.; Winter, R. *Chem. Eur. J.* **2018**, 24, 14346-14351.

Expression and purification of α-synuclein

The expression and purification of α-synuclein was carried out as described.[1-2] The plasmid was purchased from GenScript. The plasmid pT7-7 ex-pressing human α-synuclein was transformed into Escherichia coli strain BL21 (DE3). A single colony was picked and inoculated into 100 mL LB medium containing 150 μg/mL ampicillin and grown at 37 °C with shaking at 250 rpm until the absorbance at 600 reached 0.8. Induction was then carried out by adding 1 mM IPTG (final concentration) and the culture was further

grown under similar conditions for 3 h. The cells were harvested and resuspended in 0.75 mL buffer (50 mM Tris-HCl, pH 7.5, 10 mM EDTA and 150 mM NaCl) and frozen at -80 °C. Tubes containing frozen cells were placed in a boiling water bath for 7 min, and the supernatant was collected after centrifugation at 12000 rpm for 5 min. Streptomycin sulfate (136 µL/mL of supernatant) and glacial acetic acid (228 µL/mL of supernatant) were added and centrifuged for 2 min. Again, the supernatant was recovered and precipitated with ammonium sulfate (saturated ammonium sulfate at 4°C was used 1:1, v/v, with supernatant). The protein was collected as precipitate by centrifugation and washed once with 1 mL of ammonium sulfate solution (4 °C, 1:1, v/v, saturated ammonium sulfate and water). The washed pellet was resuspended in 900 µL of 100 mM ammonium acetate (to form a cloudy solution) and precipitated by adding an equal volume of ethanol at room temperature. Precipitation with ethanol was repeated once more. The pellet was resuspended in 100 mM ammonium acetate and extensively dialyzed against 10 mM Tris-HCl buffer, pH 7.4.

References

1. Volles, M.J.; Lansbury, P. T. Relationships between the sequence of α-synuclein and its membrane affinity, fibrillization propensity, and yeast toxicity. *J. Mol. Biol.* **2007**, 366, 1510-1522.

2. Shaltiel-Karyo, R.; Frenkel-Pinter, M.; Egoz-Matia, N.; Frydman-Marom, A.; Shalev, D. E.; Segal, D.; Gazit, E. Inhibiting α-Synuclein Oligomerization by Stable Cell Penetrating β-Synuclein Fragments Recovers Phenotype of Parkinson's Disease Model Flies. *PLoS One*, **2010**, 5, e13863.

Additional Figures

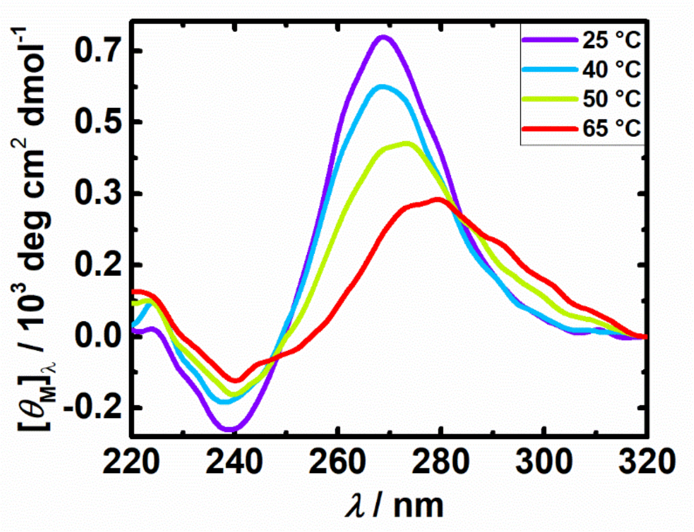

Figure S 1: CD-Spectra of the RNA core G-quadruplex sequence only (20 µM RNA). The buffer was 20 mM $Na_xH_xPO_4$, pH 7.5, with 140 mM KCl.

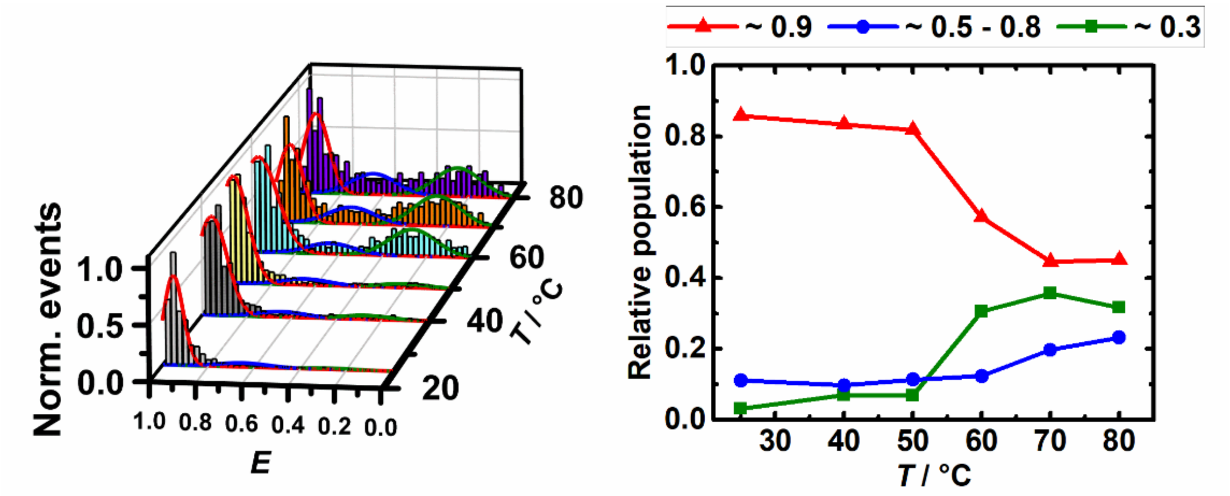

Figure S 2: FRET efficiency and relative conformational population of the elongated and labeled RG-1 RNA in 20 mM $Na_xH_xPO_4$ buffer at pH 7.5 with 140 mM KCl at different temperatures.

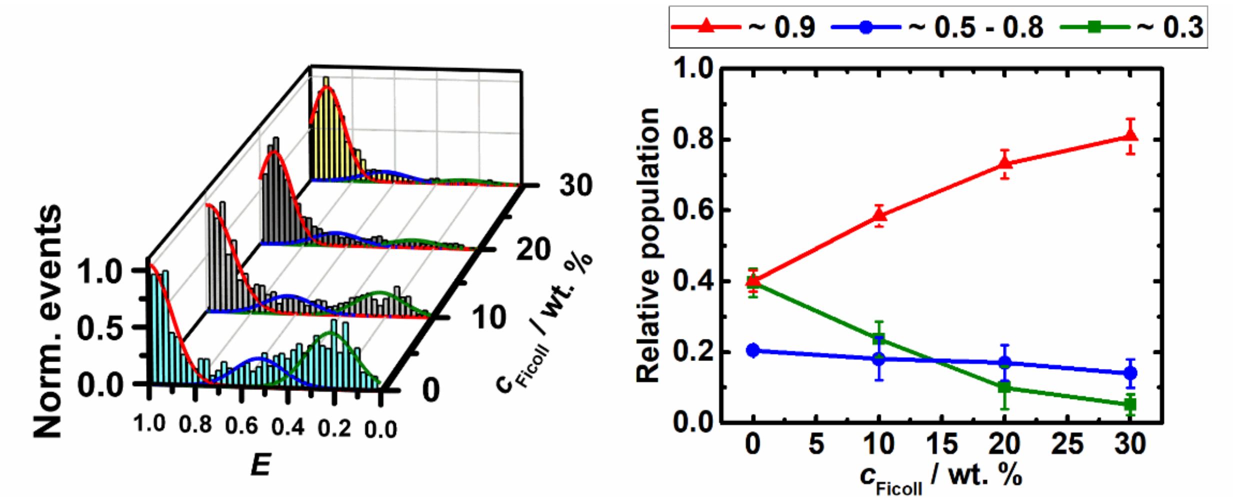

Figure S 3: FRET efficiency and relative conformational population of the elongated and labeled RG-1 RNA in 15 mM KCl, 20 mM TrisHCl buffer at pH 7.5 and 25 °C with increasing concentration of Ficoll PM 70.

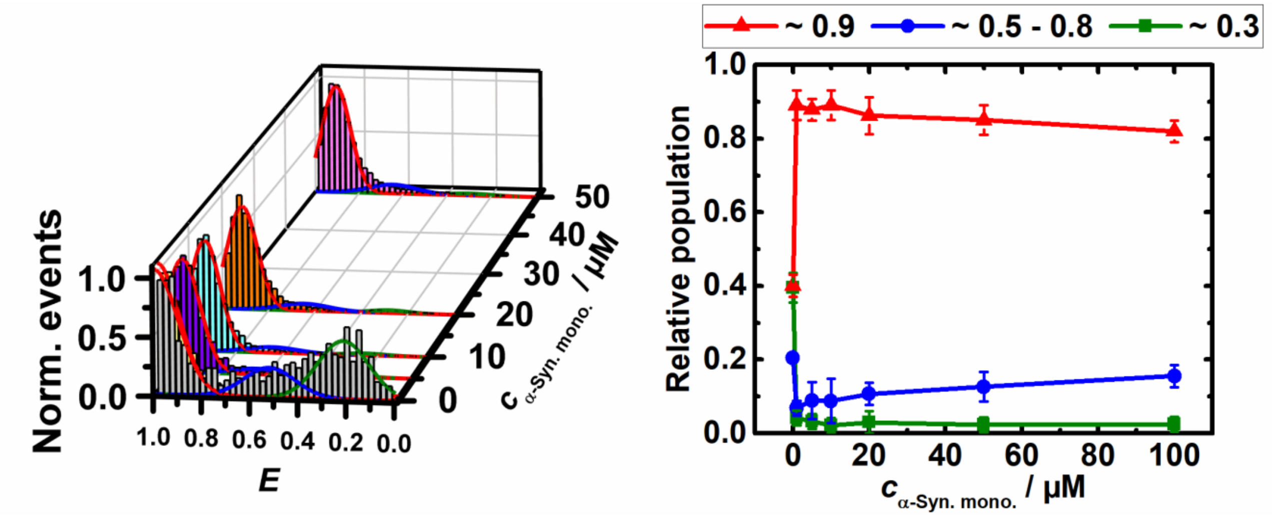

Figure S 4: FRET efficiency and relative conformational population of the elongated and labeled RG-1 RNA in 15 mM KCl, 20 mM TrisHCl buffer at pH 7.5 and 25 °C with increasing concentration of α-Syn monomers.
